# Patients with periodontitis might increase the risk of urologic cancers: a bidirectional two-sample Mendelian randomization study

**DOI:** 10.1007/s11255-023-03858-w

**Published:** 2023-11-28

**Authors:** Bojia Li, Yifei Lin, Yong Yang, Zeng Wang, Rui Shi, Tao Zheng, Banghua Liao, Ga Liao, Jin Huang

**Affiliations:** 1 Health Management Center, General Practice Medical Center, Medical Device Regulatory Research and Evaluation Center, West China Hospital, Sichuan University, Chengdu, Sichuan People’s Republic of China; 2 West China School of Public Health, West China Hospital, Sichuan University, Chengdu, Sichuan People’s Republic of China; 3 Engineering Research Center of Medical Information Technology, Ministry of Education, West China Hospital of Sichuan University, Chengdu, Sichuan People’s Republic of China; 4 Department of Urology, Institute of Urology (Laboratory of Reconstructive Urology), West China Hospital, Sichuan University, Chengdu, Sichuan 610044 People’s Republic of China; 5 State Key Laboratory of Oral Diseases, National Clinical Research Center for Oral Diseases, West China Hospital of Stomatology, Sichuan University, Chengdu, Sichuan 610041 People’s Republic of China

**Keywords:** Periodontitis, Immunity, Urologic neoplasms, Genetic epidemiology, Mendelian randomization, Causal relationship

## Abstract

**Background:**

Numerous observational epidemiological studies have reported a bidirectional relationship between periodontitis and urological cancers. However, the causal link between these two phenotypes remains uncertain. This study aimed to examine the bidirectional causal association between periodontitis and four types of urological tumors, specifically kidney cancer (KC), prostate cancer (PC), bladder cancer (BC), and testis cancer (TC).

**Methods:**

Based on large-scale genome-wide association study (GWAS) data, we utilized the two-sample Mendelian randomization (MR) approach to evaluate causal relationships between periodontitis and urological cancers. Several MR methods covering various consistency assumptions were applied in this study, including contamination mixture and Robust Adjusted Profile Score to obtain robust results. Summary-level data of individuals with European ancestry were extracted from the UK Biobank, the Kaiser GERA cohorts, and the FinnGen consortium.

**Results:**

Our findings revealed significant positive genetic correlations between periodontitis and kidney cancer (OR 1.287; 95% CI 1.04, 1.594; *P* = 0.020). We did not find a significant association of periodontitis on prostate cancer, bladder cancer, and testis cancer. In reverse MR, no significant results were observed supporting the effect of urologic cancers on periodontitis (all *P* > 0.05).

**Conclusion:**

Our study provides the evidence of a potential causal relationship between periodontitis and kidney cancer. However, large-scale studies are warranted to confirm and elucidate the underlying mechanisms of this association.

**Supplementary Information:**

The online version contains supplementary material available at 10.1007/s11255-023-03858-w.

## Introduction

Urological cancers are a heterogeneous group of neoplasms with diverse clinical and molecular characteristics, which can affect any part of the urological system. In the Cancer Statistics Report of 2022, urological cancers accounted for three of the ten most common cancers in men, namely prostate, bladder, and kidney. Prostate cancer was reported causing 34,500 deaths, ranking as the second leading cause of cancer mortality in men. Bladder and kidney cancers accounted for 6% and 5% of all new cancer cases in men, respectively. Among women, kidney cancer was one of the most common malignancies, ranking the ninth in incidence [1]. The financial burden of urological cancers in Europe was estimated to exceed €13 billion annually [2, 3]. With the rising incidence rate, urological cancers are expected to pose an increasing burden on patients and society. Unfortunately, the etiology of urologic cancers remains unclear, and more risk factors need to be explored.

The prevention of urological cancers is difficult due to their complex etiology, multiple risk factors, and lack of obvious initial symptoms [4–7]. Commonly known risk factors for urological cancers include age, genetic factors, and smoking [8]. However, periodontitis is increasingly recognized as an important risk factor for urological cancer. Several meta-analyses have demonstrated a significant association between periodontitis and an increased risk of prostate cancer, indicating that periodontitis increases the risk of urological cancers by 17–79% [9–11]. A retrospective study involving 121,240 Korean elder adults showed a significant positive association between chronic periodontal disease and prostate cancer [12]. Additionally, another study found that men with a history of periodontal disease, including periodontitis and gingival disease, had a 19% higher risk of developing invasive bladder cancer [13]. Systemic inflammation induced by urologic cancers and the potential metastasis of cancer cells can contribute to the development of oral health issues. Indeed, a number of case studies have documented instances of gingivitis being initiated in association with bladder and kidney cancers [14, 15].

Given the observed bidirectional relationship between urological cancer and periodontitis, and the presence of numerous confounding factors, scholars have posited that genetic factors may play a significant mediating role in this association. Some studies have indicated that genetic variations in specific genes may be linked to the development of both urological cancers and periodontitis [16, 17]. Nevertheless, the majority of these studies have concentrated on individual genes, and large-scale investigations are still needed to substantiate these findings. Therefore, additional research is necessary to further elucidate the causal relationship between urological cancers and periodontitis.

In recent years, Mendelian randomization (MR) has gained popularity as a robust method for causal inference in epidemiological research [18]. By leveraging genetic factors as instrumental variables, MR can provide strong evidence for causal relationships between exposures and outcomes, while minimizing the influence of confounding and reverse causation [19, 20]. Compared to traditional observational studies, MR is considered superior in its ability to infer causality. Many MR studies have been conducted to investigate factors associated with either periodontitis or urological cancers. For instance, an MR study found that alcohol consumption was positively associated with the odds of periodontitis [21], while another study suggested that genetic variants in alcohol metabolizing genes may be linked to prostate cancer mortality [22]. However, to date, no MR study has been conducted to examine the causal relationship between periodontitis and urological cancers.

Using large-scale genome-wide association studies (GWAS) of European ancestry, we conducted bidirectional two-sample MR analyses to identify the causal relationship between periodontitis and urological cancers, including kidney, testis, prostate, and bladder cancer. Our findings may offer valuable insights for future experimental validation and provide additional evidence for the etiology of urological cancers as well as facilitate better prediction of urological cancers.

## Materials and methods

### Study design and population

**Fig. 1:**
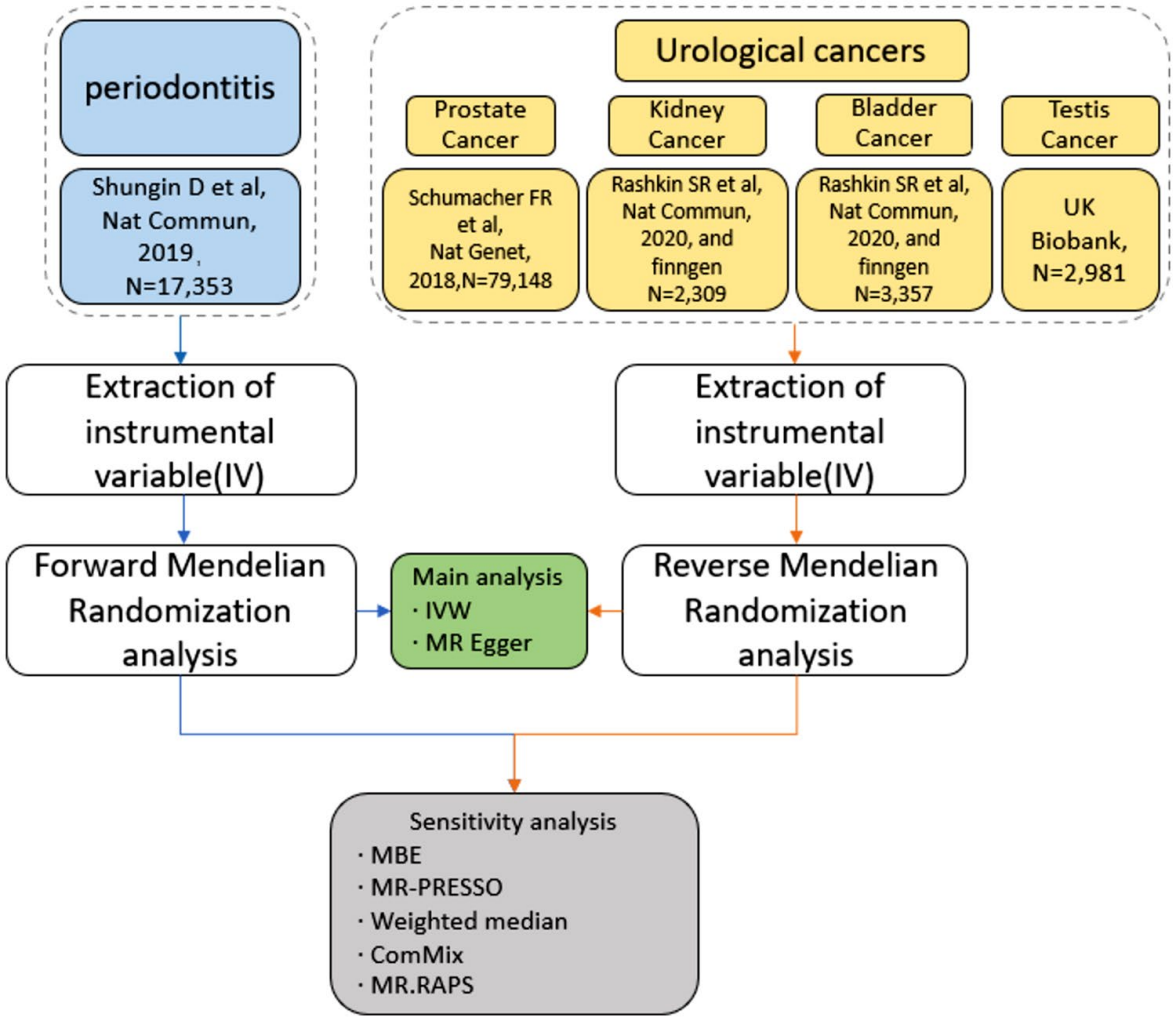
Study design and workflow

The study was performed using published summary-level data from genome-wide association studies (GWAS) of the traits of interest in predominantly European individuals. We retrieved summary statistics from the Gene–Lifestyle Interactions in Dental Endpoints (GLIDE) consortium for periodontitis (17,353 cases and 28,210 controls) [23]. We selected prostate cancer (PC) from a prostate cancer susceptibility loci study of 79,148 cases and 61,106 controls [24]. We used GWASs from the pan-cancer study of UK Biobank and Kaiser GERA cohorts (1338 cases and 408,786 controls) [25] and the FinnGen consortium for kidney cancer (KC) (971 cases and 217,821 controls). Variants associated with bladder cancer (BC) were obtained from the pan-cancer study of UK Biobank and Kaiser GERA cohorts (2242 cases and 410,350 controls) [25] and the FinnGen consortium (1115 cases and 217,677 controls). Variants associated with testicle cancer (TC) were obtained from a GWAS of 2981 cases and 401,788 controls from the White British participants of the UK Biobank. Source and sample size of GWAS summary statistics are presented in Supplementary Table 1. To improve the power of statistical analysis in the presence of a modest sample size, we conducted a meta-analysis using METAL for kidney cancer and bladder cancer [26]. We used the LiftOver tool to convert all GWAS summary data that have reference genome GRCh38/hg38 to GRCh37/hg19 to format. The flowchart of the study is presented in Fig. 1.

As this study derived summary statistics from publicly available GWASs, all ethical approvals and informed consent for the GWAS were obtained by the original GWAS authors.

### Selection of genetic variants associated with periodontitis and urinary cancers

**Fig. 2:**
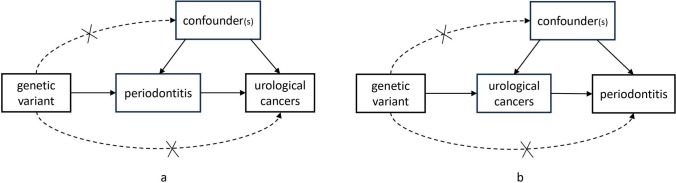
Conceptual illustration of the Mendelian Randomization (MR) method, (**a**) the forward MR, (**b**) the reverse MR

MR requires three key assumptions for instrumental variants (Fig. 2): (1) genetic variants are robustly associated with the exposure of interest; (2) variants are not associated with confounders between exposure–outcome relationships; (3) genetic variants do not affect the outcome of interest except through the exposure of interest [19, 20]. Thus, if the genetic variation affected the outcome, it could only arise through exposure, ruling out the effect of confounding factors. To meet the first hypothesis, independent SNP were selected using PLINK 1.9 (forward parameters and reverse parameters for TC and KC:— - clump- p1 5 × 10^−6^— - clump- p2 1 × 10^−5^— - clump- r2 0.01— - clump- kb 500; reverse parameters for BC and PC:— - clump- p1 5 × 10^−8^— - clump- p2 1 × 10^−5^— - clump- r2 0.01— - clump- kb 500).

### Mendelian randomization analyses

The MR analysis was performed using R studio software (version 4.0.3) with two packages, “Mendelian Randomization” and “MR-PRESSO”.

In addition to several classical MR methods (Median-based methods, Mendelian Randomization-Egger regression (MR-Egger), inverse-variance weighted (IVW), and Mode-Based Estimate (MBE)), we applied two new methods, namely, contamination mixture (ConMix) [27] and Robust Adjusted Profile Score (RAPS) [28], to investigate potential causal inferences between periodontitis and urinary cancers. The new methods can help down-weight the effect of weak instrument bias, pleiotropy, and extreme outliers. MR-Pleiotropy Residual Sum and Outlier (MR-PRESSO) methods were used for sensitivity analyses.

## Results

### The role of periodontitis in urinary cancers

**Fig. 3:**
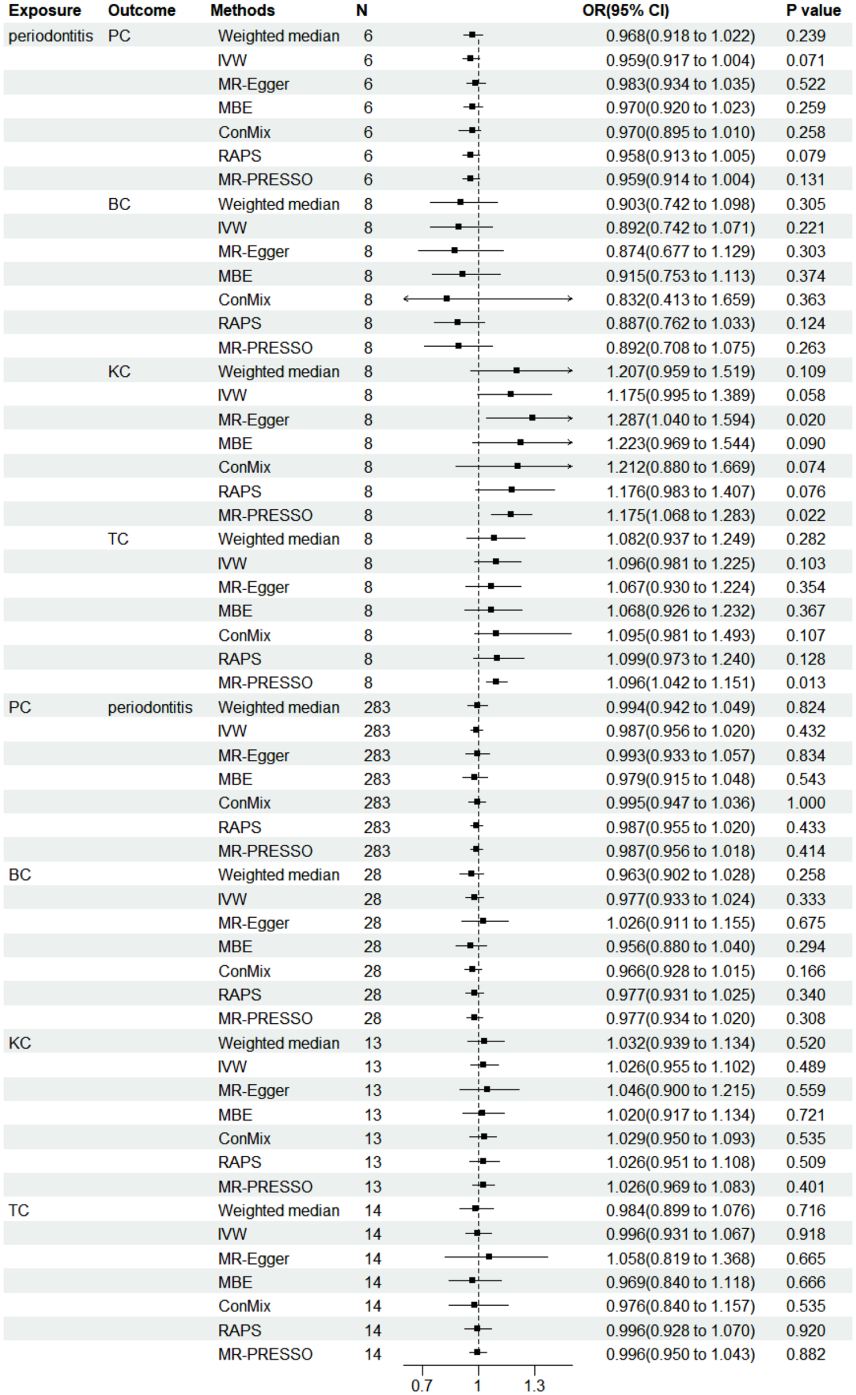
Forest plots of the MR analyses testing the causal association between periodontitis and urologic cancers

Under the primary genome-wide significance *P* value threshold of *P* < 5 × 10^−6^, a total of 6, 8, 8, and 8 instrumental SNPs were retained for PC, KC, BC, and TC, respectively. Figure 3 shows the forest plots of the MR analyses testing the causal association between periodontitis and urologic cancers.

In terms of kidney cancer, we found evidence indicating a potential causal relationship between periodontitis with KC (MR-Egger OR 1.287; 95% CI 1.04–1.594; *P* = 0.020), with the non-significant intercept (*P* = 0.180) suggesting that the pleiotropic effect can be ignored (Supplementary Table 2). The MR-PRESSO results agreed well with the results of MR-Egger (OR = 1.175, *P* = 0.022) (Supplementary Table 3). IVW (OR 1.175; 95% CI 0.995–1.389; *P* = 0.058) and RAPS (OR 1.176; 95% CI 0.983–1.407; *P* = 0.076) supported the finding marginally. MR-PRESSO did not detect potential outliers in the study.

However, we did not find convincing evidence of a causal relationship between periodontitis and BC (IVW: OR 0.892; 95%CI 0.742–1.071; *P* = 0.221), PC (IVW OR 0.959; 95% CI 0.917–1.004; *P* = 0.071), as well as TC (IVW OR 1.096; 95% CI 0.981–1.225; *P* = 0.103).

### The role of urinary cancers on periodontitis

Under the primary genome-wide significance *P* value threshold of *P* < 5 × 10^–6^ (PC using 5 × 10^–8^), 268, 8, 13 and 14 instrumental SNPs were left for PC, KC, BC, and TC, respectively. No significant results were observed supporting the significant effect of urinary cancers on periodontitis (*P* > 0.05) (Supplementary Table 2). MR-PRESSO testing casual effect of urologic cancers on periodontitis are provided in Supplementary Table 4.

## Discussion

We conducted a bidirectional two-sample Mendelian randomization (MR) analysis to investigate the causal relationship between periodontitis and four urological cancers, namely, kidney, testicular, prostate, and bladder cancer. Our study utilized large-scale genome-wide association studies (GWAS) summary data from over 400,000 individuals of European ancestry, as well as the latest MR methods.

In the forward analysis, we found a significant causal relationship between periodontitis and kidney cancer using the IVW, MR-Egger, and MR-PRESSO method. Although we obtained heterogeneous results, our results are still reliable. Below are two of the reasons:

First, different MR methods have different assumptions and principles, as well as their own strengths and weaknesses. When directional pleiotropy is absent, the IVW method can deliver a relatively stable and accurate causal evaluation using a meta-analytic approach to combine Wald estimates for each IV [29, 30]. The MR-Egger method’s estimate is known to be relatively robust to the presence of pleiotropy [31]. However, compared to the IVW method, other methods like the MBE have compromised power, which may exhibit negative results, especially with limited SNPs [32]. Consequently, the IVW was used as the primary method to evaluate causality in our study, and other methods served as complementary methods.

Second, current studies have also reported the significant causal inference when obtaining heterogeneous results. A study testing the causal association between Insulin-Like Growth Factor 1 levels and migraine risk (Abuduxukuer R et al.) reported a positive result in spite of 8 out of 19 MR ORs are non-significant [33]. Wootton et al. [34] reported that there is a significant association between smoking initiation and the risk of developing schizophrenia, while the ORs derived from the MR-Egger and Weighted Mode method are deemed statistically non-significant (the ORs obtained from the IVW, Weighted Median, and RAPS methods were statistical significance). Wei et al. [35] reported a causal association between birthweight and latent autoimmune diabetes in adults using the weighted median method and MR-Egger yielded insignificant results (the IVW, Robust IVW, and MR-PRESSO methods have significant results).

Thus, although not all MR methods were significant, it is reasonable to conclude that periodontitis is causally associated with kidney cancers.

However, in the reverse analysis, no causal effect of the four genetically predicted urological cancers on periodontitis was observed. This can serve as a reference for future clinical decision-making and experimental validation.

Our findings regarding the positive association between periodontitis and kidney cancer are consistent with current research. A longitudinal study spanning 18 years and involving 48,375 male healthcare professionals in the United States revealed that individuals with a history of periodontal disease exhibited a 49% higher risk of developing kidney cancer [36]. Another study reported that periodontal disease slightly increases the risk of kidney cancer [37]. Given that some studies have not found any association between periodontitis and kidney cancer [37, 38], our results provided important complementary evidence supporting the positive causal link between periodontitis and kidney cancer. Particularly, the evidence presented in this study is reliable, as it is unlikely to be influenced by confounding factors.

In terms of prostate cancer, our study did not find evidence for the hypothesis that periodontitis may be causally associated with the risk of prostate cancer. However, several studies have investigated the potential relationship between periodontitis and its incidence. For example, a study conducted in Korea reported a 14% increase in the prevalence of prostate cancer among men with periodontitis [31], whereas a Swedish study observed a 47% increase [32]. Similarly, two Turkish studies also found a significant correlation between periodontitis and prostate cancer [39, 40]. However, some other studies claimed not to observe such an association [38, 41, 42], and two of them even reported a negative correlation between tooth loss (which can result from periodontitis) and prostate cancer [36, 43].

Similarly, although our study did not establish a causal relationship between periodontitis and bladder cancer, the current literature supports a potential association between the two. For instance, a study with over 700,000 participants found that periodontitis was associated with a 30.7% increased risk of bladder cancer [44]. Another study reported a significant 13–45% higher risk of bladder cancer associated with periodontitis [41]. Besides, there was a positive but statistically insignificant correlation between periodontitis and bladder cancer in four other studies [36, 37, 41, 45]. What’s more, a study has reported no significant association between periodontal disease and genitourinary malignancies in postmenopausal women [37].

We propose that the discrepancies in results may be due to the small sample size in our study or the small number of genetic instruments. Moreover, differences in diagnostic criteria for periodontitis may have influenced the outcomes. Furthermore, the presence of horizontal pleiotropy could potentially introduce bias if the single-nucleotide polymorphisms (SNPs) under consideration exhibited associations with confounding factors via pathways unrelated to periodontitis. Additionally, bias might be introduced in cases where the relationships between the exposure and the outcome departed from linearity, and there was insufficient available data for thoroughly investigating such deviations. Nonetheless, further studies are required to elucidate the exact causal relationship between periodontitis and testicular and bladder cancer.

A multitude of potential mechanisms have been proposed to explicate the causal linkage between periodontitis and urological cancers. The influence of periodontitis on urological cancer is thought to be mediated by systemic chronic inflammation, shared risk factors, oral pathogenic microorganisms, impaired immune function, and genetic susceptibility. First, periodontitis is a chronic inflammatory condition that may result in systemic inflammation [46], a known contributor to cancer development, as evidenced by the therapeutic use of anti-inflammatory drugs in certain cancer types [47, 48]. Second, oral dysbiosis, an important pathogenic mechanism of periodontitis [49], may be associated with the development of urological cancers due to the presence of oral pathogenic microorganisms in the genitourinary system. Finally, shared genetic factors may partly explain the effect of periodontal disease on urogenital cancers. However, the precise mechanisms underlying the relationship between periodontitis and urological cancers remain uncertain, and further research is needed to fully elucidate the role of periodontitis in the initiation of urological cancers.

Our results have a considerable clinical significance. As a potential causal risk factor of urological cancers, periodontitis should be highly concerned in preventing urological cancers. Clinicians should consider incorporating periodontal evaluation and treatment into the routine care of cancer patients, as periodontitis may negatively impact their treatment outcomes and life quality of patients. On the other hand, when caring for patients with periodontitis, the risk of developing urological cancers should be assessed and early intervention could be made. Most importantly, periodontitis could be taken into account as a risk factor in future studies on the etiology of urologic tumors, whether in observational, clinical, or genetic studies.

The strengths of this study are as follows. First, this is the first MR study reporting the causal relationship between periodontitis and urological cancers, which has been receiving widespread attention. Second, the study used two-sample MR framework for causal inference, excluding the effect of confounding factors. We employed several MR methods, including two novel approaches, ConMix and RAPS, to enhance the reliability and reduce the potential for bias in our findings. Despite these strengths, our study is not without limitations. Our study is subject to certain limitations. Despite utilizing newly developed MR methods and the latest available GWAS data, our study's sample size for cases was relatively small, which may have resulted in limited statistical power. Therefore, future studies with larger sample sizes are required to validate our findings. Second, the study population was predominantly composed of individuals of European descent, and thus, the generalization of our results to other ethnic groups should be made with caution.

In conclusion, our results provide evidence identifying a causal relationship between periodontitis and KC, whereas we did not find evidence to support the causal relationship between periodontitis and PC, TC, and BC. That is of vital importance for better managing patients with periodontitis clinically. While no evidence was found supporting the causal effect of urological cancers on periodontitis. The exact mechanism of the causal relationship between periodontitis and urological cancers deserves further investigation.

### Contribution to the field statement

Urological cancers are a group of neoplasms that are known to have varying clinical and molecular characteristics, affecting the entire urological system. According to recent cancer statistics, these cancers are anticipated to impose an increasing burden on both patients and society. The prevention of urological cancers is challenging, owing to their intricate etiology, multiple risk factors, and lack of conspicuous initial symptoms. Consequently, it is of paramount clinical importance to determine the underlying causes of urological cancers. Among the various risk factors associated with these cancers, periodontitis has been widely recognized as a significant contributor. Previous studies have reported on the potential association between periodontitis and urological cancers, yet the causal effect has remained unproven. In light of this, we applied a genetic approach known as Mendelian randomization to evaluate the causal relationships between periodontitis and urological cancers. Through this approach, which utilizes large-scale GWAS summary data, we expected to offer strong evidence for the causal link between exposures and outcomes. Our findings revealed significant positive genetic correlations between periodontitis and kidney cancer, testis cancer, as well as prostate cancer. Our findings have significant implications for future research and offer valuable guidance to clinicians. Clinicians can now screen patients with periodontitis for early cancer, particularly those with common urological cancer risk factors, and in the elderly population. Early screening and diagnosis may aid in reducing the risk of cancer progression.

### Supplementary Information

Below is the link to the electronic supplementary material.

Supplementary file1 (DOCX 29 KB)

## Data Availability

All data analyzed during the current study are publicly available. The source of data can be found in supplementary table 1.
