# Supplementary material for: Patients with periodontitis might increase the risk of urologic cancers: a bidirectional two-sample Mendelian randomization study
Source: Int Urol Nephrol. 2023 Nov 28;56(4):1243–51. doi: 10.1007/s11255-023-03858-w (PMC10923993; doi:10.1007/s11255-023-03858-w)
Supplement: Supplementary file 1 — Supplementary file1 (DOCX 29 KB) [file 11255_2023_3858_MOESM1_ESM.docx]

Supplementary Table 1. Source and sample size of GWAS summary statistics

| **Phenotype** | **Abbreviation** | **Data source** | **case** | **control** | **Sample size** | **Reference genome** | **Imputation panel** | **# SNPs** |
| --- | --- | --- | --- | --- | --- | --- | --- | --- |
| periodontitis | periodontitis | Genome-wide analysis of dental caries and periodontitis combining clinical and self-reported data. Nat Commun, 2019 | 17353 | 28210 | 17353 | GRCh37 | 1000G | 28210 |
| prostate cancer | PC | Association analyses of more than 140,000 men identify 63 new prostate cancer susceptibility loci. Nat Genet, 2018 | 79148 | 61106 | 79,148 | GRCh37 | 1000G | 61106 |
| kidney cancer | KC | Pan-cancer study detects genetic risk variants and shared genetic basis in two large cohorts. Nat Commun, 2020 | 1338 | 408786 | 1,338 | GRCh37 | UK10K and 1000 G | 408786 |
|  |  | gs://finngen-public-data-r5/summary_stats/finngen_R5_C3_KIDNEY_NOTRENALPELVIS.gz | 971 | 217821 | 971 | GRCh38 | SISu v3 | 217821 |
| bladder cancer | BC | Pan-cancer study detects genetic risk variants and shared genetic basis in two large cohorts. Nat Commun, 2020 | 2242 | 410350 | 2,242 | GRCh37 | UK10K and 1000 G | 410350 |
|  |  | gs://finngen-public-data-r5/summary_stats/finngen_R5_C3_BLADDER.gz | 1115 | 217677 | 1,115 | GRCh38 | SISu v3 | 217677 |
| testicle cancer | TC | https://pheweb.org/UKB-SAIGE/pheno/187.2 | 2981 | 401788 | 2,981 | GRCh37 | HRC | 401788 |

Supplementary Table 2. Results of the MR analyses testing the causal association between periodontitis and urologic cancers

| Exposure | Outcome | MR Method | Number of instrument | OR | 95% CI | p-value |
| --- | --- | --- | --- | --- | --- | --- |
| *The forward MR analyses* | | | | | | |
| periodontitis | prostate cancer | Weighted median | 6 | 0.968 | 0.918,1.022 | 0.239 |
|  |  | IVW |  | 0.959 | 0.917,1.004 | 0.071 |
|  |  | MR-Egger |  | 0.983 | 0.934,1.035 | 0.522 |
|  |  | (intercept) |  | -0.011 | -0.024,0.002 | 0.089 |
|  |  | MBE |  | 0.970 | 0.92,1.023 | 0.259 |
|  |  | ConMix |  | 0.970 | 0.778,0.895 | 0.258 |
|  |  | RAPS |  | 0.958 | 0.913,1.005 | 0.079 |
| periodontitis | bladder cancer | Weighted median | 8 | 0.903 | 0.742,1.098 | 0.305 |
|  |  | IVW |  | 0.892 | 0.742,1.071 | 0.221 |
|  |  | MR-Egger |  | 0.874 | 0.677,1.129 | 0.303 |
|  |  | (intercept) |  | 0.006 | -0.042,0.054 | 0.812 |
|  |  | MBE |  | 0.915 | 0.753,1.113 | 0.374 |
|  |  | ConMix |  | 0.832 | 0.413,1.659 | 0.363 |
|  |  | RAPS |  | 0.887 | 0.762,1.033 | 0.124 |
| periodontitis | kidney cancer | Weighted median | 8 | 1.207 | 0.959,1.519 | 0.109 |
|  |  | IVW |  | 1.175 | 0.995,1.389 | 0.058 |
|  |  | MR-Egger |  | 1.287 | 1.04,1.594 | 0.020 |
|  |  | (intercept) |  | -0.028 | -0.07,0.013 | 0.180 |
|  |  | MBE |  | 1.223 | 0.969,1.544 | 0.090 |
|  |  | ConMix |  | 1.212 | 0.88,1.669 | 0.074 |
|  |  | RAPS |  | 1.176 | 0.983,1.407 | 0.076 |
| periodontitis | testicle cancer | Weighted median | 8 | 1.082 | 0.937,1.249 | 0.282 |
|  |  | IVW |  | 1.096 | 0.981,1.225 | 0.103 |
|  |  | MR-Egger |  | 1.067 | 0.93,1.224 | 0.354 |
|  |  | (intercept) |  | 0.010 | -0.02,0.04 | 0.509 |
|  |  | MBE |  | 1.068 | 0.926,1.232 | 0.367 |
|  |  | ConMix |  | 1.095 | 0.981,1.493 | 0.107 |
|  |  | RAPS |  | 1.099 | 0.973,1.24 | 0.128 |
| *The reverse MR analyses* | | | | | | |
| prostate cancer | periodontitis | Weighted median | 283 | 0.994 | 0.942,1.049 | 0.824 |
|  |  | IVW |  | 0.987 | 0.956,1.02 | 0.432 |
|  |  | MR-Egger |  | 0.993 | 0.933,1.057 | 0.834 |
|  |  | (intercept) |  | -0.001 | -0.006,0.004 | 0.820 |
|  |  | MBE |  | 0.979 | 0.915,1.048 | 0.543 |
|  |  | ConMix |  | 0.995 | 0.947,1.036 | 1.000 |
|  |  | RAPS |  | 0.987 | 0.955,1.02 | 0.433 |
| Bladder cancer | periodontitis | Weighted median | 28 | 0.963 | 0.902,1.028 | 0.258 |
|  |  | IVW |  | 0.977 | 0.933,1.024 | 0.333 |
|  |  | MR-Egger |  | 1.026 | 0.911,1.155 | 0.675 |
|  |  | (intercept) |  | -0.010 | -0.033,0.013 | 0.387 |
|  |  | MBE |  | 0.956 | 0.88,1.04 | 0.294 |
|  |  | ConMix |  | 0.966 | 0.928,1.015 | 0.166 |
|  |  | RAPS |  | 0.977 | 0.931,1.025 | 0.340 |
| kidney cancer | periodontitis | Weighted median | 13 | 1.032 | 0.939,1.134 | 0.520 |
|  |  | IVW |  | 1.026 | 0.955,1.102 | 0.489 |
|  |  | MR-Egger |  | 1.046 | 0.9,1.215 | 0.559 |
|  |  | (intercept) |  | -0.006 | -0.044,0.033 | 0.774 |
|  |  | MBE |  | 1.020 | 0.917,1.134 | 0.721 |
|  |  | ConMix |  | 1.029 | 0.95,1.093 | 0.535 |
|  |  | RAPS |  | 1.026 | 0.951,1.108 | 0.509 |
| testicle cancer | periodontitis | Weighted median | 14 | 0.984 | 0.899,1.076 | 0.716 |
|  |  | IVW |  | 0.996 | 0.931,1.067 | 0.918 |
|  |  | MR-Egger |  | 1.058 | 0.819,1.368 | 0.665 |
|  |  | (intercept) |  | -0.009 | -0.048,0.029 | 0.633 |
|  |  | MBE |  | 0.969 | 0.84,1.118 | 0.666 |
|  |  | ConMix |  | 0.976 | 0.84,1.157 | 0.535 |
|  |  | RAPS |  | 0.996 | 0.928,1.07 | 0.920 |

Supplementary Table 3. MR-PRESSO testing casual effect of periodontitis on urologic cancers

| **Exposure** | Outcome | **MR Analysis** | **Causal Estimate** | **Sd** | **T-stat** | **P-value** | **GlobalTest.RSSobs** | **GlobalTest.Pvalue** |
| --- | --- | --- | --- | --- | --- | --- | --- | --- |
| **periodontitis** | **prostate cancer** | Raw | -0.042 | 0.023 | -1.805 | 0.131 | 6.980 | 0.583 |
|  |  | Outlier-corrected | NA | NA | NA | NA |  |  |
|  | **bladder cancer** | Raw | -0.115 | 0.094 | -1.224 | 0.260 | 13.551 | 0.263 |
|  |  | Outlier-corrected | NA | NA | NA | NA |  |  |
|  | **kidney cancer** | Raw | 0.162 | 0.055 | 2.938 | 0.022 | 3.566 | 0.913 |
|  |  | Outlier-corrected | NA | NA | NA | NA |  |  |
|  | **testis cancer** | Raw | 0.092 | 0.028 | 3.322 | 0.013 | 2.897 | 0.950 |
|  |  | Outlier-corrected | NA | NA | NA | NA |  |  |

Supplementary Table 4. MR-PRESSO testing casual effect of urologic cancers on periodontitis

| **Exposure** | Outcome | **MR Analysis** | **Causal Estimate** | **Sd** | **T-stat** | **P-value** | **GlobalTest.RSSobs** | **GlobalTest.Pvalue** |
| --- | --- | --- | --- | --- | --- | --- | --- | --- |
| **prostate cancer** | **periodontitis** | Raw | -0.013 | 0.016 | -0.818 | 0.414 | 262.088 | 0.812 |
|  |  | Outlier-corrected | NA | NA | NA | NA |  |  |
| **bladder cancer** |  | Raw | -0.023 | 0.022 | -1.040 | 0.308 | 24.948 | 0.711 |
|  |  | Outlier-corrected | NA | NA | NA | NA |  |  |
| **kidney cancer** |  | Raw | 0.025 | 0.029 | 0.871 | 0.401 | 8.412 | 0.877 |
|  |  | Outlier-corrected | NA | NA | NA | NA |  |  |
| **testis cancer** |  | Raw | -0.004 | 0.024 | -0.151 | 0.882 | 6.689 | 0.946 |
|  |  | Outlier-corrected | NA | NA | NA | NA |  |  |
